# Inflammatory Bowel Disease: Complexity and Variability Need Integration

**DOI:** 10.3389/fmed.2018.00075

**Published:** 2018-03-21

**Authors:** Claudio Fiocchi

**Affiliations:** ^1^Department of Pathobiology, Lerner Research Institute, Cleveland, OH, United States; ^2^Department of Gastroenterology and Hepatology, Digestive Disease and Surgery Institute, Cleveland Clinic, Cleveland, OH, United States

**Keywords:** inflammatory bowel disease, ulcerative colitis, Crohn’s disease, systems biology, network medicine, complexity, variability

In the last century, humanity has being increasingly affected by a large number of chronic conditions that fall into two major categories: neoplastic and inflammatory diseases. Unlike infectious diseases, which have a defined etiological agent, the etiology of these two dominant forms of disease is still undefined, although there is overwhelming evidence that the cause is multifactorial as are the underlying biological mechanisms (1). Chronic inflammatory diseases, autoimmune or otherwise, exhibit two cardinal features: complexity and variability (2). Complexity is due to the seemingly endless number of factors and mechanisms associated with the disease, and variability is due to the intrinsic heterogeneity of the affected individuals and the surrounding environment in which they live in. Complexity and variability are inextricably intertwined in conditioning, predisposing, triggering, and mediating the disease process and the ensuing clinical manifestations, which are also variable and unpredictable. These features are shared by numerous conditions but, in this opinion article, the focus will be on inflammatory bowel disease [IBD; Crohn’s disease (CD) and ulcerative colitis (UC)] as the prototypical example of a chronic inflammatory disease (3). The main factors that contribute to the complexity and variability of IBD will be discussed, the need to integrate these factors will be highlighted, and comprehensive data integration offered as the solution to the development of target-specific therapies that allow the implementation of precision medicine.

Physicians responsible for the care of IBD patients are used to the inconsistency of the clinical manifestations they observe, the unpredictable outcome of the chosen therapeutic intervention, and the need for long-term monitoring to try to anticipate and prevent flare-ups. A logical explanation for this lack of consistency is that whatever the underlying mechanism(s) of disease is in one patient is not the same in another patient, so that the common failure of “one size fits all” therapy should not come as a surprise. Clinical variation results from biological variation of the factors implicated in disease pathogenesis (3). We currently accept the environment, the genetic makeup, the gut microbiota, and the immune response as the “main factors” involved in predisposing to and mediating IBD, but this interpretation increasingly appears to be a naïve and unrealistic simplification of a much more complex and multifactorial process (4). The environment is essentially unlimited and constantly changes in response to human behavior and evolution (5–7); the number of genes is limited, but they contain millions of single-nucleotides polymorphisms (SNPs) as well as other variants in addition to displaying abundant pleiotropy (8), and genes represent a mere 1–2% of the genome which is subject to the regulatory action of the remaining 98% of the genome and of gene–gene interactions (9); the gut microbiota contains trillions of bacteria in addition to the gut virome and mycobiome, all of them interacting and functionally affecting each other (10–12); the immune system, which is the actual effector arm that mediates inflammation and causes tissue damage in IBD, is relatively small in cell type composition, but its secreted products (cytokines, chemokines, reactive species, etc.) tremendously expand its biological capacity concomitantly with their dynamic and mutually influencing interactions (13). Facing this overwhelming complexity, how can we possibly learn what is going on in any individual patient at the biological and molecular level and then rationally choose the best treatment option? History, physical examination, lab tests, imaging, endoscopy, and biopsies can simply confirm that we are dealing with some form of IBD of a certain location, extent, and clinical severity, but these routine tests tell us essentially nothing about subtle environmental exposures, rare genetic abnormalities, composition and dynamics of the gut microbiota, and the specific inflammatory pathways involved. So, facing the powerlessness of this reality, we end up treating the patients with a series of non-specific anti-inflammatory or antimicrobial drugs hoping for a positive response, even knowing that response will be only partially effective and of limited duration (14), after which subsequent rounds of the same approach will follow with generally similar results. This article will limit the discussion to the biological complexity of IBD, which is only a tiny component of the much larger complexity of solving health-care issues in the population at large (15).

New drugs, small molecules, or biologics for IBD are under constant development, but they are created in a relative knowledge vacuum of the real and extraordinary complexity of IBD pathogenesis. All new compounds will benefit some subgroup of patients, but we cannot anticipate which ones, how effective they will be and for how long (16). While continuing this therapeutic approach is justifiable at the moment because of the need to strive for better and more diverse forms of treatment, it is obvious that brand new ideas and tactics are needed. The first step in fulfilling these goals is to accept the reality that complex diseases require complex therapies, a fact long acknowledged in the field of oncology. Treatments based on single drugs, as it is still commonly done in IBD, will never achieve optimal results because the molecular malfunctions underlying the disease differ from patient to patient and undergo multiple changes over the course of the disease (17). The second step is to accept biological complexity and acknowledge patient variability (2, 18). Biological complexity and individual variability occur at all levels of the host. Just to cite a few examples, protein levels are heritable molecular phenotypes that exhibit considerable variation between individuals, populations, and sexes (19); epigenetic modifications, such as DNA methylation, vary substantially among human tissues (20); and genetic variants cause deregulations that are highly specific to disease-relevant cell types or tissues (21). After admitting biological complexity and acknowledging patient variability, which only requires a change in mindset and the will of taking on a new challenge, one should then ask the question of how to address these issues (22). This is indeed a major challenge, one that the medical community alone is not ready nor capable of taking on alone, and one that requires both acquiring new knowledge and look for new partnerships (21).

The previously mentioned pathogenic factors associated with IBD, i.e., the environment, the genes, the microbiota, and the immune systems, may be indeed critically important but, given their individual complexity, they must be studied and analyzed adopting brand new tools. Most critically, all of them, and whatever new factors may be uncovered in IBD pathogenesis, must be functionally integrated (13, 18, 23–25). Only after achieving such integration one can then zero on the key molecules mediating the disease in each patient and achieve the much desired personalized therapy for CD and UC patients (26). Two major steps must be taken to accomplish such a lofty but essential goal: (1) identify and analyze pathogenic factors in their totality and (2) integrate knowledge from all combined totalities.

The analysis of factors in their totality can be accomplished with an “omic” approach, an “ome” being defined as the totality of any particular complex system (27). This approach creates the field of “omics,” i.e., the study of all omes implicated in IBD (or any other complex disease), such as exposomics (exposome being a synonym for environment), genomics, metagenomics, and immunomics, as well as all other omes probably involved in IBD, including epigenomics, transcriptomics, proteomics, metabolomics, and so on (6, 7, 24, 28, 29). The number of omics is expanding so rapidly that is becoming a whole new world in itself (30). Exploring this new world will uncover massive amount of data whose analysis will require computational approaches to qualify and quantity, which brings up the need to apply systems biology methodologies (31). Systems biology can be simply defined as the computational modeling of complex biological systems (*Wikipedia*), or an approach in biomedical research to understanding the larger picture by putting its pieces together, in stark contrast to decades of reductionist biology, which involves taking the pieces apart (*NIH/NIAID*). Alternatively, systems biology can be defined as the science of integrating genetic, genomic, biochemical, cellular, physiological, and clinical data to create networks to model predictively disease expression and response to therapy (31). In other words, system biology allows us to practically look into and analyze vast quantities of information whose volume and complexity go way beyond the capacity of the human mind.

Once IBD-relevant omes are recognized and evaluated in their totality, the next task is to understand how they interact among each other (32), how they are regulated, and what controls the key biological events responsible for the disease process (33, 34), i.e., intestinal inflammation in the case of CD or UC. This task is the essence of network medicine (35, 36) and also requires computational approaches (32). Quoting from the recently published Network Medicine book, “*Rather than trying to force disease pathogenesis into a reductionist model, network medicine embraces the complexity of multiple influences on disease and relies on many different types of networks. By developing techniques and technologies that comprehensively assess genetic variation, cellular metabolism, and protein function, network medicine is opening up new vistas for uncovering causes and identifying cures of disease*” (37). In other terms, network medicine uses well established methods that are commonly utilized in other, non-biological complex systems, such as computer networks, airline networks, financial networks, or social networks. In every network, there are controlling elements driving that network, and when such controlling elements are eliminated, the network is disrupted and no longer capable to exert its function (38). Typical examples are the breakdown of a server that will incapacitate all connected computers, or the disruption of a single hub controlling thousands of flights with the consequent collapse of an airline network. Translating these examples into biological terms, once the controller(s) of a network responsible for a disease such as IBD is identified, such controller becomes the target of specific intervention with the subsequent disintegration of the network, i.e., the elimination of the disease process (34).

The process of assembling biological regulatory networks starts with measurements of all disease-relevant omics by collecting multiple biosamples from multiple sources at several time points during the course of the disease, and submitting the data to computational analysis to create a regulatory network (24), also called an interactome (39). This is formed by a large number of nodes (molecules) and a much smaller number of hubs (key regulatory nodes) that control the network, all of them being connected through edges (24, 39). Construction of the interactome can start with a seed gene selection derived from existing databases such as genome-wide associated studies, Online Mendelian Inheritance in Man, and current literature followed by algorithms designed to identify a particular molecular neighborhood of the network that represents the “disease module,” which is then validated based on gene expression data, gene ontologies, other pathways, etc. (24). This allows to arrive to a biological interpretation of the disease module and the prioritization of the most important pathway(s) and the identification of the molecular targets that control the whole disease network (the disease interactome). This is then followed by high throughput discovery screening to match drugs existing in compound databases with the molecular targets identified in the disease interactome. A system biology-based approach to develop and target the IBD interactome has been recently reported (4).

The above sequence of steps obviously requires far more than an excellent clinical setting; it also requires a state-of-the-art bioinformatics setting staffed by computational biologists knowledgeable in disease processes. Therefore, to solve complex diseases such as IBD, integration must occur not only at the biological level but also at the professional level (40). Medicine is becoming increasingly dependent on multiple technologies, and the amount of information that can be derived from examining a patient in great detail will soon exceed the analytical capacity of even the most knowledgeable and experienced clinician (41). To achieve the most precise diagnosis, define the clinical phenotype, and choose the most appropriate form of therapy for any given IBD patient, the so-called precision medicine approach will require to integrate routine clinical and laboratory data with environmental, genetic, epigenetic, transcriptional, proteomic, metabolomic, microbial, and immune data. This task is beyond the capacity of any medical professional, and artificial intelligence support will become progressively indispensable (42). In 1970, an article predicted that by the year 2000 computers would act as a powerful extension of the physician’s mind and decision-making, but this would require solving major intellectual and technical problems (43). In 2018, it is obvious that many of these problems have been solved while others are well under way of being solved, and artificial intelligence is ready to handle the unprecedented and continuously growing amount of data generated by basic, translational and clinical studies, and wearable health sensors (44). A practical example of how artificial intelligence can be exploited to aid in clinical medicine is in the finding of optimal drug doses (45). Using a personalized dosing platform, represented by a second-order algebraic equation with experimentally determined coefficients of the equation unique to each subject, liver transplantation patients have significantly less variability in tacrolimus trough levels compared with control patients receiving physician-guided dosing (46). Thus, it is increasingly evident that traditional medicine will not be able to ever satisfactorily address all the demands of complex diseases, and integration of medical and computational knowledge is mandatory to achieve real progress in the diagnosis and management of IBD or, for that matter, of any chronic inflammatory disease.

## Author Contributions

The author confirms being the sole contributor of this work and approved it for publication.

## Conflict of Interest Statement

The author declares that the research was conducted in the absence of any commercial or financial relationships that could be construed as a potential conflict of interest.
